# Dysplasia in oral lichen planus: relevance, controversies and challenges. A position paper

**DOI:** 10.4317/medoral.24610

**Published:** 2021-06-20

**Authors:** Miguel Ángel González-Moles, Saman Warnakulasuriya, Isabel González-Ruiz, Ángela Ayén, Lucía González-Ruiz, Isabel Ruiz-Ávila, Pablo Ramos-García

**Affiliations:** 1 School of Dentistry, University of Granada, Granada, Spain; 2 Instituto de Investigación Biosanitaria ibs. Granada, Granada, Spain; 3 WHO Collaborating Group for Oral Cancer; 4 Faculty of Dental, Oral and Craniofacial Sciences, King's College London, London, UK; 5 Dermatology Service, San Cecilio Hospital Complex, Granada, Spain; 6 Dermatology Service, Ciudad Real General University Hospital, Ciudad Real, Spain; 7 Pathology Service, San Cecilio Hospital Complex, Granada, Spain

## Abstract

**Background:**

Patients with oral lichen planus (OLP) have an increased risk of oral cancer. For this reason, OLP is classified as an oral potentially malignant disorder. However, the precise personal (or individual) risk is unknown. Recent meta-analytical studies have reported that dysplastic OLP may transform to cancer in around 6% of cases, while the rate of transformation is lower (<1.5%) in non-dysplastic cases. The presence of epithelial dysplasia has emerged as the most powerful indicator for assessing cancer risk in oral potentially malignant disorders in routine practice. However, the general acceptance of epithelial dysplasia as an accompanying histologic feature in OLP is subject to great controversy. Many pathologists consider the presence of dysplasia as a criterion to exclude OLP when routinely reporting on this disease. This practice, widespread among oral pathology professionals, has resulted in the underestimation of the potential for malignancy of OLP.

**Material and Methods:**

A review of the literature was carried out in order to critically analyze the relevance, controversies and challenges encountered across the diagnosis of epithelial dysplasia in OLP.

**Results:**

12 studies have been published examining dysplastic changes in OLP, reporting Figures ranging from 0.54% to 25% of cases with dysplasia in the first diagnostic biopsy. The diagnosis of dysplasia in the OLP poses an additional difficulty due to the fact that the affected oral epithelium per se develops changes related to autoimmune aggression. Among the most frequent histological features of OLP that develops dysplasia are basal cell hyperplasia with basaloid appearance, loss of basal cells polarity, cellular and nuclear pleomorphism and irregular stratification.

**Conclusions:**

Epithelial dysplasia should not be considered an exclusion criterion for OLP; its evaluation requires experienced pathologists in this field.

** Key words:**Oral lichen planus, epithelial dysplasia, oral cancer.

## Introduction

**Figure 1:**
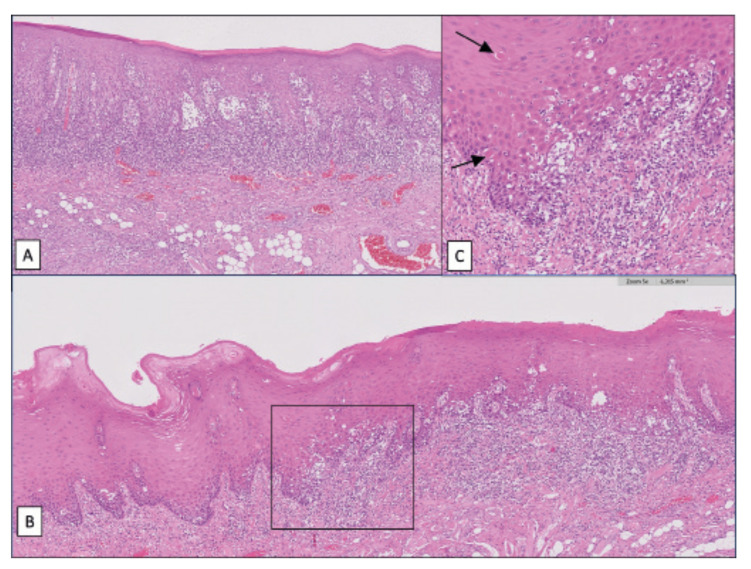
Two images of oral mucosa showing epithelial dysplasia are presented. A) belongs to an OLP lesion; B) corresponds to an oral epithelium in a patient without OLP in whom epithelial dysplasia has developed lichenoid events, especially a band-like inflammatory infiltrate, Civatte bodies, liquefaction degeneration of the basal layer and interface mucositis phenomena; C) These aspects can be seen in this figure which corresponds to an enlargement of the black-framed space in figure b. Black arrows are pointing to Civatte bodies. The difficulty in evaluating epithelial dysplasia in OLP advises considering, in addition to the histological presentation, the data from the patient's medical history and the clinical appearance of the lesions.

Oral lichen planus (OLP) is currently classified as an oral potentially malignant disorder (OPMD) (1,2) with a global prevalence of around 1% (3). Several systematic reviews and meta-analyses have estimated the ratios for malignant transformation of OLP ranging from 0.44-2.28% (1,4,5). On the aspects related to malignant transformation of OLP, mainly the presence of epithelial dysplasia has generated considerable controversy (6). The origin of the aforementioned controversy arose from the Krutchkoff and Eisenberg’s research published several decades ago (7) who, meticulously reviewed a case series of OLP that transformed to cancer, established that the initial biopsy did not correspond with a diagnosis of OLP, but represented erroneous diagnoses of other lesions that clinically could appear as a mixture of red and white lesions showing epithelial dysplasia. They argued that, in response to dysplasia that was observed microscopically, these cases could have developed lichen-like histological events sometime later, essentially an inflammatory lichenoid infiltrate. Those lesions were categorized by Krutchkoff’s group as an entity called “oral lichenoid ​​dysplasia”, a term that is at the center of the controversy. The authors recommended that all “atypical” cases of OLP presenting with epithelial dysplasia should be excluded from studies examining the risk of malignancy of this lesion. Surprisingly, with a snowball effect and without further scientific evidence, this exclusion criterion was accepted by most experts reporting on OLP; the presence of epithelial dysplasia in a biopsy was established as an exclusion criterion in diagnostic pathology when reporting on OLP, i.e. if epithelial dysplasia is present, it is not OLP (8-10). The available evidence raises a key question regarding whether or not the detection of epithelial dysplasia invalidates the diagnosis of OLP. Nevertheless, the experience of many clinicians and pathologists shows that some cases of OLP clinically well documented may develop epithelial dysplasia along the course of its evolution (11), and this opinion is also shared by the authors of this paper (Fig. 1). The consideration of epithelial dysplasia as a criterion for excluding a diagnosis of OLP has resulted in misclassification of more serious OLP cases leading to a remarkable underestimation of the potential for malignant transformation of this disease (1,12).

## Material and Methods

A review of the literature was carried out in order to meticulously analyze the implications of epithelial dysplasia in OLP. We searched MEDLINE/PubMed -as main electronic database- and Web of Science -for bibliometric analysis purposes- for studies published before the year 2020 (upper limit), with no lower date limit. Searches were conducted by combining thesaurus terms used by the databases (i.e., MeSH) with free terms, constructed to maximize sensitivity. In a first line general search, the root keywords combined were “oral lichen planus”, “oral lichenoid lesions”, “oral potentially malignant disorders” and “epithelial dysplasia”. In addition, several more specific searches were conducted combining relevant aspects of the subsections to be reviewed (physiological molecular regulation, biological functions, oncogenic mechanisms, diagnostic and prognostic implications, oral oncogenesis, and therapeutic implications). We also manually screened the reference lists of retrieved studies for additional relevant studies. Most of the studies were included or excluded according to an exhaustive analysis of the title, abstract, year of publication, impact of the journal and number of citations received. Although these last two criteria may introduce a potential selection bias, its application is necessary when handling a large number of records (e.g., in this context, simply searching: ("Lichen Planus, Oral"[mh] OR "oral lichen planus"[all] OR "olp"[tiab] OR "oral lichenoid lesion"[all] OR "oll"[tiab] OR (“potentially”[all] AND “malignant”[all] AND disorder*[all]) OR “Mouth Neoplasms”[mh] OR malign*[all] or premalign*[all] or "Carcinoma, Squamous Cell"[mh] or "oscc"[tiab] or “transformation”[tiab] or "risk"[tiab] or "progression" [tiab]) AND (“dysplasia”[all]), more than 20,000 registers were retrieved).

## Results

Twelve studies have been published examining dysplastic changes in OLP (13-23), reporting Figures ranging from 0.54% to 25% of cases with dysplasia in the first diagnostic biopsy, only one of them (24) did not find dysplasia. The diagnosis of dysplasia in the OLP poses an additional difficulty due to the fact that the affected oral epithelium per se develops changes related to autoimmune aggression. Among the most frequent histological features of OLP that develops dysplasia are basal cell hyperplasia with basaloid appearance, loss of basal cells polarity, cellular and nuclear pleomorphism and irregular stratification. In addition, to discuss some controversial aspects on the subject we have used at the discretion of the authors, when necessary other research papers that did not appear in the search strategy.

## Discussion

- Molecular basis of epithelial dysplasia in OLP

The concept of oral epithelial dysplasia refers to an alteration of the morphology of the oral epithelium, detecTable under an optical miscroscopic observation in tissue samples stained with hematoxylin-eosin, reflecting a disturbance of the epithelial architecture coupled with the presence of cellular atypia. Epithelial dysplasia reveals a disordered epithelial maturation with an increase in cell proliferation. Currently, epithelial dysplasia is considered as the gold-standard for the evaluation of the cancer progression risk of an OPMD (2). These morphological changes occur because of development of genomic instability, i.e. the cell’s predisposition to accumulate mutations and/or other genomic alterations affecting oncogenes and tumor suppressor genes, essentially as the consequence of an increased proliferative activity (25). Some molecular findings in OLP are common to those that develop in epithelial dysplasia found in other OPMD (26). Among them, stands out the loss of heterozygosity (LOH), an alteration affecting the function of both alleles of a gene. LOH reaches its greatest importance when it affects the 3p and 9p chromosomal loci (27), where several important tumor suppressor genes (25) are located. LOH consequently leads to the loss of regulatory function of the cell cycle, favoring the cell to enter in an uncontrolled proliferation state with genomic instability, development of new oncogenic summative events and contributing to a high risk of malignancy (25). Zhang *et al*. (27) reported that while OLP without dysplasia exhibits LOH in 6% of cases, in OLP with epithelial dysplasia LOH appears in more than 40 % of cases. These proportions are close to their findings in others dysplastic OPMD without lichenoid events. The authors conclude that OLP provides a fertile field for the development of dysplasia. The term "oral lichenoid dysplasia" has been used but this is confusing and should not be used. The features actually correspond to OLPs that have developed epithelial dysplasia in their evolution (28). The study of DNA ploidy has been used to assess the risk of malignant transformation of OLP, with promising results (28). It has also been reported in some studies (29) a significant association between aneuploidy and the presence and severity of epithelial dysplasia in OPMD, among which is OLP, indicating that the abnormal total nuclear DNA content may be a molecular event of epithelial dysplasia.

**Figure 2:**
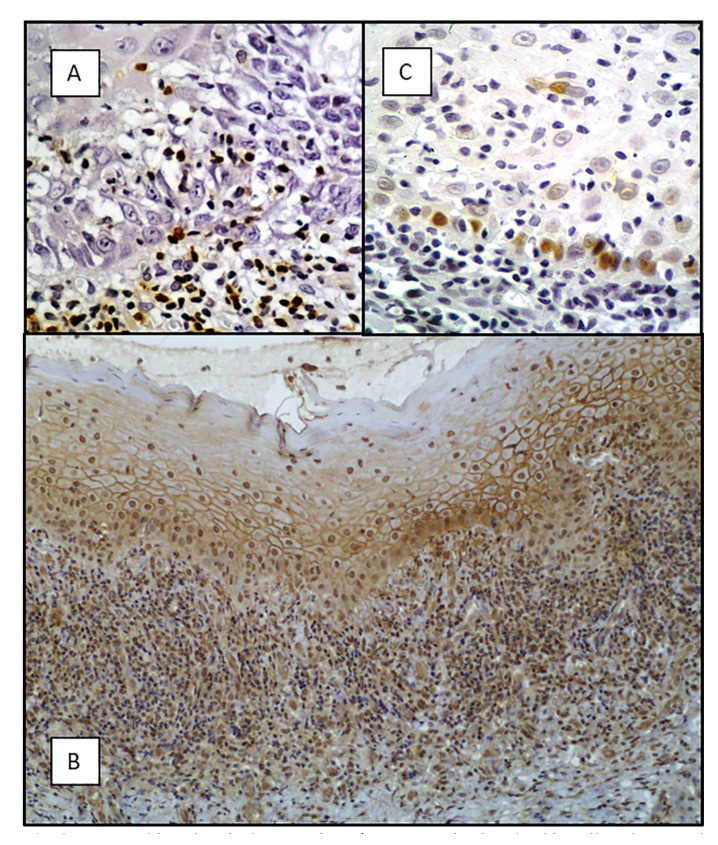
Immunohistochemical expression of some proteins involved in cell cycle control in OLP: A) expression of the anti-apoptotic protein Bcl-2 in the OLP inflammatory infiltrate; B) expression of the pro-proliferative protein Substance P; C) expression of the proliferation marker Ki-67 in the basal layer of the epithelium in OLP.

Data on immunohistochemical studies examining the expression of proteins involved in the cell cycle control, most of them encoded by tumor suppressor genes, also support the possibility that OLP may develop dysplasia through its evolution. Alterations in the function of cell cycle repressor proteins p53, CDKN1A, p16, CDK4 acquire great relevance as links in the transition towards malignancy of the OLP epithelium (30-32). Furthermore, an overexpression of the anti-apoptotic protein Bcl-2 in OLP epithelium (30,31) and OLP-associated infiltrating lymphocytes have also been demonstrated by immunohistochemical techniques (Fig. 2), which constitute a mechanism for maintaining autoimmune aggression (32). Overexpression of survivin, which acts as an apoptosis inhibitor, has also been reported in OLP (33). Moreover, down-regulation of apoptosis promoters like caspase-3 and Fas/FasL in OLP have been reported (32,34,35). Finally, activation of the pro-proliferative pathway PI-3K (33) together with some proteins involved in this pro-proliferative pathways, as substance *P* and its receptor NK-1R (Fig. 2) (35), have been also demonstrated in the OLP epithelium. Many of these molecular mechanisms are probably activated as a consequence of actions linked to aggression mediated by the inflammatory infiltrate as it has been described with reference to nuclear factor kappa beta (NF kappa B), which is overexpressed in inflammatory processes and also in cancer; also reported in head and neck squamous cell carcinomas and in OLP, where NF kappa B is related to the cytotoxic activity of the inflammatory infiltrate (36). Finally, matrix metalloproteinases proteins (MMP), especially MMP-2 and -9 have been identified in OLP where it appears to play a role associated with the disruption of the basement membrane, with the death of keratinocytes, and with the process of exocytosis. Some other researchers have reported an increase in cell proliferation markers (Fig. 2), i.e. expression -PCNA, Ki-67, cyclin D1- in OLP (32,34,35). We hypothesize (32) that this epithelial hyperproliferative response together with the suppression of apoptosis represents a mechanism for regeneration of the damaged oral epithelium, as an attempt to avoid its loss of healing in erosive OLP, the most severe form of the disease. However, the epithelium must pay a high price for establishing this protective hyperproliferative mechanism: the development of dysplasia with genomic instability and the risk of malignancy.

- The role of chronic inflammation

In recent years, the idea that chronic inflammation is an important risk factor for cancer is receiving significant attention, in this sense OLP is not an exception (37). Thus, infiltrating lymphocytes and their chemokines can be a powerful cancer promoting factors (Fig. 1). It is known that the macrophage inhibitory factor, RANTES chemokine and cycloxygenase-2 (COX-2) could be responsible for the apoptosis inhibition and for the increase in cell proliferation observed in OLP. In addition, COX-2 can also exert mutagenic effects on epithelial cells through the induction of its carcinogenic metabolite malondialdehyde, derived from the involvement of COX-2 in the arachidonic acid metabolism (32). As a consequence of chronic inflammation, oral epithelium affected by lichen planus is exposed to mutagenic stimuli. The antiapoptotic mechanisms mentioned earlier, with loss of the function of some tumor suppressor genes related with LOH, and other mutagenic factors in the inflammatory infiltrate, constitutes a field change for the development of epithelial dysplasia and therefore risk of cancer.

- Frequency of presentation and difficulty in recognizing dysplastic changes in OLP

The study of the presence of dysplasia in OLP has been essentially limited, as previously mentioned, by the widespread consideration of epithelial dysplasia as a diagnostic exclusion criterion of the disease (8,38). To date, 12 studies have been published examining dysplastic changes in OLP, only one of them (24) did not find dysplasia while the remaining 11 studies reported Figures ranging from 0.54% to 25% of cases with dysplasia in the first diagnostic biopsy (13-23); It is interesting to note that some authors (22) have reported a significant increase in the frequency of epithelial dysplasia in sequential biopsies, with 12.9% exhibiting dysplasia in the first biopsy and 48% in OLP cases in a subsequent biopsy; the authors note the second biopsy was taken to assess the worsening of the clinical appearance. A recent retrospective regional study from Autralia that traced OLP cases in their cancer registry noted that only OLP cases that had dysplasia in the original biopsy reports had later developed malignancy (19). From these data it can be deduced that epithelial dysplasia is a likely phenomenon in OLP. Further studies and a revision of the diagnostic criteria of the disease are required to establish the true prevalence of this important risk marker for cancer progression. However, the evaluation of the presence of dysplasia and assigning a grade for dysplasia in our view needs a thorough discussion among the experts working in this field. The diagnosis of dysplasia in OLP poses an additional difficulty due to the fact that the affected oral epithelium per se develops changes related to autoimmune aggression - vacuolizing degeneration of the basal layer of the epithelium, Civatte bodies, interphase mucositis, etc.- that could be confused with some histologic features of dysplasia; this is especially relevant in cases of mild dysplasia in which the changes are confined to the lower third of epithelium, where epithelial histological abnormalities (8) in OLP are mainly observed. This observation is further supported by studies reporting most frequently lichenoid histological events in mild-moderate dysplasia, and that lichenoid morphology disappears as the severity of the dysplasia increases (39), probably as a consequence that the histological appearance of severe dysplasia/carcinoma in situ is so conclusive that do not allow the pathologist another alternative diagnosis. The studies that evaluate the frequency of specific histological events of dysplastic OLP (8,21,23) have shown that they primarily affect the basal epithelial layer - basal cell hyperplasia with basaloid appearance, loss of basal cells polarity, cellular and nuclear pleomorphism, irregular stratification -all of them could easily be confused with some characteristic features of OLP (Table 1)-. It has also been pointed out (39) that even been the band-like inflammatory infiltrate one of the most common and easily recognizable lichenoid ​​events, the difficulty in its interpretation is based on the non-specific nature of a phenomenon that develops in epithelia expressing antigens, as commonly occurs in the oral mucosa. In addition, pathologists frequently assume histological changes in epithelia attacked by inflammatory infiltrates as reactive, downplaying the importance they really deserve (36). Interface mucositis, is also a frequent misinterpreted lichenoid histological appearance, since it represents a common aspect to immune responses in the oral epithelium. Civatte bodies, which correspond to apoptotic keratinocytes, are easily confused with dyskeratotic cells (39). Another added difficulty stems from the known fact that epithelial dysplasia dramatically modifies the architecture and histological appearance of the oral epithelium; so, the question is what remains of the histology of OLP once the dysplasia has been established, developed and progressed? (1). Due to the discussed complexities, OLP should be histologically evaluated by experienced pathologists in this field, preferably by two agreeing with consensus, and the final consideration of a case as a dysplastic OLP should be the consequence of the weighted evaluation of the data of the clinical history, clinical presentation as presented by the clinician and histological evaluation. Often a MDT style clinicopath conference between the two specialists - a clinician and pathologist- may help to resolve these conflicting issues.

**Table 1:**
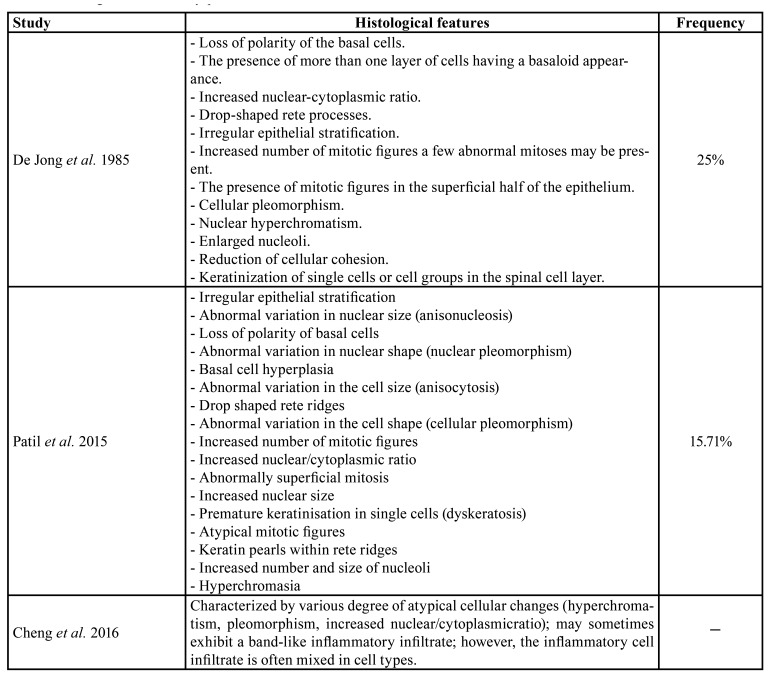
Histological features of dysplastic OLP.

- Importance of dysplasia in the malignant transformation of OLP

The meta-analytical results recently published by our research group are, in this regard, of relevance (1,4). Thus, although non dysplastic OLP may progress to cancer ranging from 0.44-2.28% of the cases (1,4,5), our analysis showed significantly higher ratios of malignancy in OLP with dysplasia (6.22%) (1). We reported that the malignant transformation ratio of OLP has probably been underestimated by the exclusion of cases with dysplasia. Further studies are warranted to accurately assess the importance of dysplasia in the malignant transformation of this disease. In our meta-analysis we also noted that studies in which patients were followed for longer periods, reported significantly higher rates of malignancy vs. those that followed their patients for shorter periods or did not communicate their follow up periods; it is logical that dysplasia can appear at any time during the evolution of OLP. Some authors continue defending in recent papers (20) a disease category under the term "lichenoid dysplasia"; Although this creates controversy and disagreement, some positive aspects must also be recognized, such as to reinforce the concept of the potentially malignant nature of OLP. From our point of view, the dysplastic appearance in the context of a lichenoid tissue response has in itself the value in risk prediction indicating or pointing towards an increased risk of oral cancer development in an individual patient. This fact should be considered as the most relevant of the process.

- Future research

By studying sequential biopsies available in pathology archives, it should be possible for the researchers to systematically examine whether epithelial dysplasia arises in established cases of OLP, particularly among cases where the first biopsy demonstrated classical features of OLP without dysplasia. Prospective studies could also be planned in centers systematically following up OLP cases for evaluating the development of epithelial dysplasia during disease evolution. As the presence of epithelial dysplasia dramatically modifies the architecture and histological appearance of the oral epithelium, we propose to develop a web based atlas as an educational resource and for networking among oral pathologists. We propose to conduct a delphi style expert opinion poll among oral pathologists to make a formal assessment of criteria when reporting on OLP.

## Conclusions

Here we present why epithelial dysplasia should not be considered as an exclusion criterion of OLP diagnosis; the diagnostic criteria for this disease should be reviewed taking into consideration the possibility of developing dysplasia and its association with the malignant transformation. OLP should be followed for long periods, probably for life, and investigated by biopsy with the main objective of assessing the presence of epithelial dysplasia. The evaluation of epithelial dysplasia in OLP, due to its complexity, requires pathologists with experience in this field taking into account the data from the patient's history and the clinical presentation of the lesions.
